# Mid- term results of stryker® scorpio plus mobile bearing total knee arthroplasty

**DOI:** 10.1186/1758-2555-4-38

**Published:** 2012-10-18

**Authors:** Hideo Kobayashi, Naoto Mitsugi, Yuichi Mochida, Naoya Taki, Yasushi Akamatsu, Masato Aratake, Hirohiko Ota, Katsushi Ishii, Kengo Harigane, Taichi Ideno, Tomoyuki Saito

**Affiliations:** 1 Department of Orthopaedic Surgery, Yokohama City University Medical Center, 4-57 Urafune-cho, Minami, Yokohama, Kanagawa, 232-0024, Japan; 2 Center for Rheumatic Diseases, Yokohama City University Medical Center, 4-57 Urafune-cho, Minami, Yokohama, Kanagawa, 232-0024, Japan; 3 Department of Orthopaedic Surgery, Yokohama City University Graduate School of Medicine, 3-9, Fukuura, Kanazawa, Yokohama, Kanagawa, 236-0004, Japan

**Keywords:** Total knee arthroplasty, Mobile- bearing design, Osteoarthritis, Rheumatoid arthritis

## Abstract

**Background:**

The mobile bearing knee system was introduced to lessen contact stress on the articular bearing surface and reduce polyethylene wear. The purpose of the current study was to investigate the mid-term results of patients undergoing total knee arthroplasties (TKAs) using Scorpio Plus Mobile Bearing Knee System (Stryker, Mahwah, NJ), and compare the outcomes between patients with osteoarthritis and osteonecrosis (OA·ON group) and patients with rheumatoid arthritis (RA group).

**Methods:**

Eight males and 58 females were followed up for a period of 4.4- 7.6 years from June 1, 2003 to December 31, 2005. There were 53 knees with osteoarthritis, 17 knees with rheumatoid arthritis, and 6 knees with osteonecrosis. Clinical and radiographic follow- up was done using The Japanese Orthopedic Association knee rating score (JOA score) and Knee Society Total Knee Arthroplasty Roentgenographic Evaluation and Scoring System.

**Results:**

With regard to the JOA score, there was significant improvement in both groups. The postoperative range of motion was between 0.8°and 116.8° in OA·ON group, and between 0.0° and 113.7° in RA group. There were no significant differences with the radiographic evaluation between two groups. Spontaneous dislocation of a polyethylene insert occurred in one patient, and deep infection was occurred in one patient.

**Conclusion:**

There was significant improvement with regard to the clinical and radiographic results of patients undergoing TKAs using the model. The risk of polyethylene insert dislocation related to the mobile bearing TKA is a cause for concern.

## Introduction

Total knee arthroplasty (TKA) is a safe and effective procedure designed to improve function and relieve the pain associated with osteoarthritis (OA), rheumatoid arthritis (RA), osteonecrosis (ON), and other types of arthritis. The mobile bearing knee system was introduced to lessen contact stress on the articular bearing surface and reduce polyethylene wear. Theoretically, by allowing tibial motion relative to the polyethylene insert, the mobile bearing knee system can improve knee flexibility and kinematics [1].

**Figure 1:**
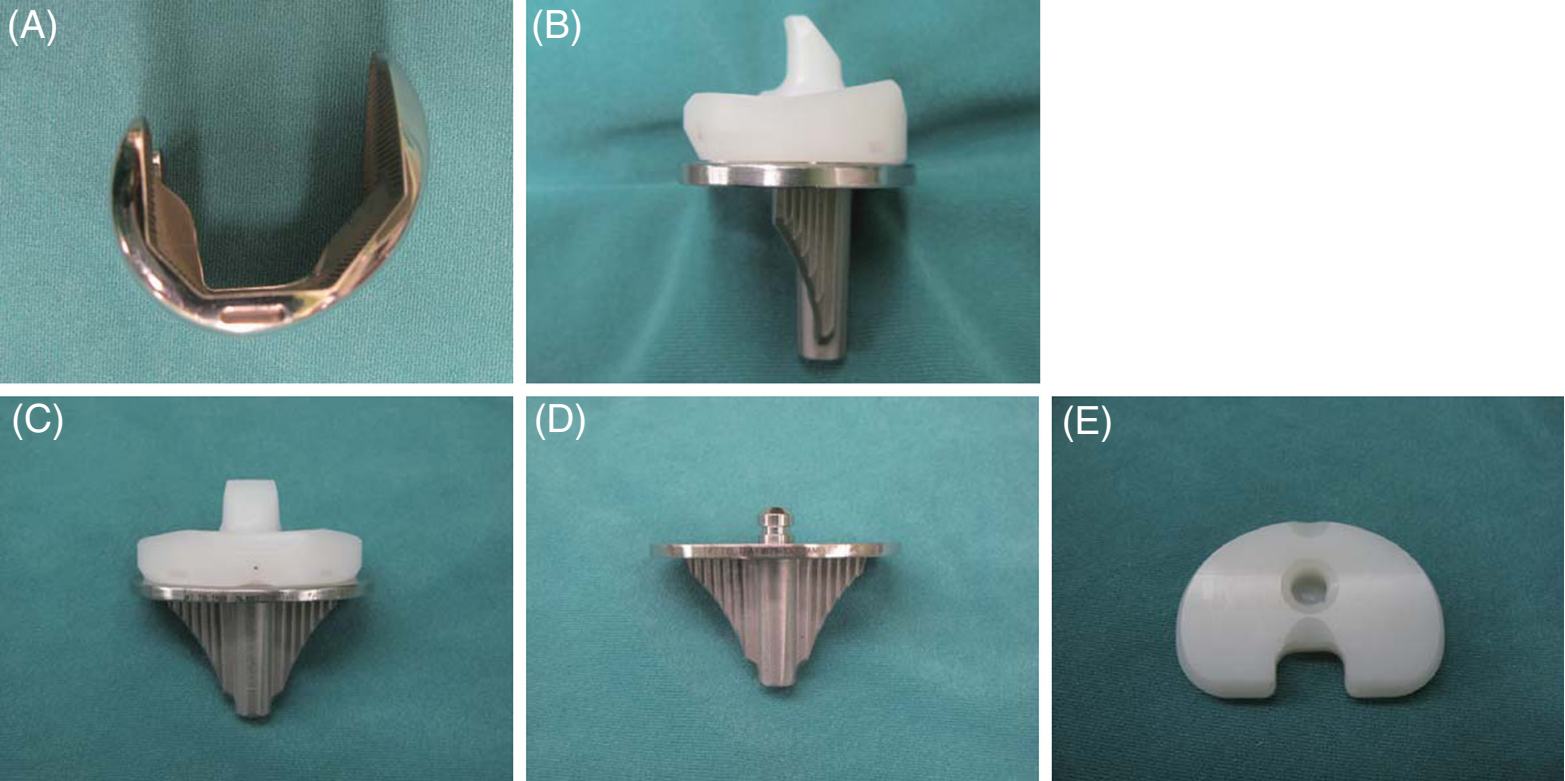
Photographs of the Scorpio Plus SuperFlex posterior- stabilized mobile bearing knee system showing the lateral (A, B) and the anterior (C, D) views and the mobile bearing surface with locking ring (E).

The Scorpio Plus SuperFlex posterior- stabilized (PS) mobile bearing knee system (Stryker, Mahwah, NJ) has been designed to provide natural rollback, increased implant conformity, and deep flexion. The femoral model has adopted a single antero- posterior and medio- lateral radius design, resulting in joint stability and quadriceps efficiency. A lowered posterior lip of the polyethylene insert lessens excess tension of both tibial and fibular collateral ligament and does not disturb deep flexion (Figure 1A, 1B). The model makes use of a mushroom-shaped post as part of the metal tibial tray which engages with the polyethylene insert and the mechanism allows unlimited internal and external rotation of the insert on the tibial articular surface (Figure 1C- 1E). On the other hand, other types of mobile systems including the low contact stress prosthesis (LCS, DePuy) and press-fit condylar prosthesis (PFC, DePuy) utilize a central cone as part of the polyethylene insert which engages a matching conical cavity in the tibial tray. Thus, mechanism of rotation is quite different among the various manufacturers of mobile- bearing TKAs. Although many studies have been performed to investigate clinical results of mobile bearing TKAs and similar clinical outcomes have been reported between mobile bearing and fixed bearing TKAs [2,3], the differences of design, shape, and mechanism of rotation might bring different clinical and radiographic results. Over 100,000 TKAs using the Scorpio mobile bearing system have been performed all over the world. However, to the best of our knowledge, no one has reported the clinical and radiographic results of the model. In addition, there are few reports assessing clinical result of mobile bearing TKAs in patients with RA [4]. Medial/ lateral laxiety or anteroposterior instability of patients with RA might result in worse clinical results compared with results of patients with OA. The purpose of the study was to investigate the mid- term results of the Scorpio PS mobile bearing TKA and to compare results between patients with OA and ON (OA·ON group) and patients with rheumatoid arthritis (RA group).

## Materials and methods

### Patients

113 primary TKA operations on 101 patients were done using Scorpio mobile bearing system between June 2003 and December 2005. Thirty two patients (34 knees) were lost to follow-up and 3 patients (3 knees) had died prior to follow- up investigation. Finally, 76 primary TKA operations on 66 patients were enrolled in this study. This study was approved by the Institutional Review Board, and all patients provided informed consent to participate in it. Fifty-three knees had OA, six knees had ON, and seventeen knees had RA. There were 8 male and 58 female patients ranging in age from 51 to 82 years. Demographic data are given in Table 1. The mean age at the operation and the preoperative femora- tibial angle (FTA) were significant different between two groups.

**Table 1:** Preoperative clinical data on patient subgroups

	**OA·ON group**	**RA group**
Patient No.	49	17
TKA No.	59 OA:53, ON:6	17
Sex (Male/ Female)	7/ 42	1/ 16
Age at time of operation (yr)	72.2±6.1 *	62.8±8.1 *
	51- 82	52- 79
Body height (cm)	149.8±6.9	151.7±6.9
Body weight (kg)	55.8±9.8	57.8±10.9
Body-mass index (kg/m^2^)	24.8±3.6	25.1±4.6
Preoperative Flexion angle (°)	120.3±18.7	109.4±24.2
Preoperative Extension angle (°)	−6.6±8.1	−15.3±12.4
Preoperative JOA score	51.6±8.9	44.1±10.9
Preoperative FTA (°)	184.9±7.9 *	177.2±7.2 *
Follow- up duration (yr)	5.8±0.8	6.1±0.9

* = *P*- values less than 0.01 by Mann- Whitney’s *U* test.

### Surgical technique

A midline or lateral curved surgical incision with a midvastus approach was used. After osteophytes were removed, the anterior cruciate ligament and posterior cruciate ligament were sacrificed. The modified gap technique was used to accomplish the accurate soft- tissue balance. Briefly, the distal femoral osteotomy was perpendicular to the mechanical axis and the proximal tibial osteotomy was perpendicular to the tibial axis in the coronal and sagittal plane. After the initial bone cuts, the soft- tissue release was performed. A balancer or spacer block was used to evaluate soft- tissue balances between the distal femur and the proximal tibia or between the posterior femur and the proximal tibia at both 0°extension and 90°flexion. All components were fixed with cement with resurfacing of the patella. Full weight-bearing and range of motion (ROM) exercises were begun from postoperative day 1.

### Clinical and radiographic evaluation

Preoperative and postoperative clinical evaluations were performed using the Japanese Orthopaedic Association osteoarthritis or rheumatoid arthritis knee rating score (JOA score) [5]. In the score, pain on walking, pain on ascending and descending the stairs, ROM, and joint swelling were rated for OA and ON patients with maximum scores of 30, 25, 35, and 10 points, respectively. Similarly, pain, ROM, manual muscle testing of quadriceps, ability of walking, and ability of ascending and descending the stairs were rated for RA patients with maximum scores of 40, 12, 20, 20, and 8 points, respectively. ROM was assessed using goniometer measurement. For radiographic evaluation, preoperative and postoperative radiographs of a standing anteroposterior radiograph and a lateral radiograph were utilized to assess FTA, the angle of components [6].

### Statistical analysis

Preoperative clinical data, preoperative and postoperative JOA score, ROM, and radiographic parameters were compared between two groups using Mann- Whitney’s *U* test. *P*- values less than 0.01 were considered statistically significant.

## Results

### Clinical results

**Figure 2:**
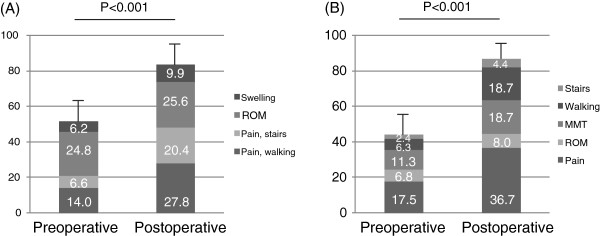
Preoperative and Postoperative JOA score of the OA·ON group (A) and the RA group (B).

The median follow- up period was 5.8±0.8 years (range 4.4–7.6 years). With regard to the JOA score, there was significant improvement in both groups; i.e. from 50.7 points to 83.6 points in OA·ON group, and from 44.1 points to 87.1 points in RA group (Figure 2). The postoperative ROM was between 0.8° and 116.8° in OA·ON group, and between 0.0° and 113.7° in RA group. No significant difference was found in the postoperative JOA score and ROM between OA·ON group and RA group (Table 2). Spontaneous dislocation of a polyethylene insert occurred in one patient with ON, and she had undergone operation. Operative findings revealed failure of the locking ring and the original insert was replaced with a thicker insert. Deep infection was occurred in one knee 18 months after surgery. The infection was treated with debridement and antibiotics therapy, and the revision arthroplasty was successfully performed 23 months after surgery.

**Table 2:** **Postoperative clinical and radiographic results of the OA**·**ON group and the RA group**

	**OA·ON group**	**RA group**
Postoperative Flexion angle (°)	116.8±16.2	113.7±16.9
Postoperative Extension angle (°)	−0.8±2.5	0.0±0.0
Postoperative JOA score	83.6±12.6	87.1±8.9
α angle	96.7±2.6	96.7±2.0
β angle	88.6±2.5	88.3±2.3
γ angle	6.3±4.1	5.2±4.5
δ angle	86.2±2.7	87.1±2.2
Postoperative FTA (°)	175.6±4.3	175.1±2.6

### Radiographic results

There were no significant differences with regard to the radiographic evaluation between two groups (Table 2).

## Discussion

Carothers et al. [2] have performed a meta- analysis study of 3506 mobile- bearing TKAs. In relation to 1880 rotating platform mobile- bearing TKAs, survival rates at 15 years (96.4%), increase of the mean Knee Society Score (62.6 points), mean final flexion (116.6°), and polyethylene dislocation (1.0%) have been reported, and they have concluded that the mobile- bearing TKA had demonstrated excellent results. To compare results of a mobile- bearing TKA with those of a fixed- bearing TKA, which have provided durable long term fixation and excellent survival rates, many authors have made a direct comparison on the use of mobile- bearing and fixed- bearing TKAs in the same patients [4,7-11]. Although Price et al. [8] reported that the American Knee Society Score, the Oxford Knee Score, and pain scores for the mobile- bearing TKA were slightly better than those for the fixed- bearing device, most authors have concluded that there were no differences in both clinical and radiographic results between two designs [4,7,9-11]. As well, a meta- analysis studies have suggested no significant differences in clinical or radiological outcomes, such as Knee Society Scores, Hospital for Special Surgery scores, ROM, and radiographic alignment between mobile- bearing and fixed- bearing TKA [12,13].

Mid- term clinical and radiographic results of the model were equivalent to results of other types of mobile- bearing TKAs described in literatures. Although a lowered posterior lip of the polyethylene insert was considered to allow deep flexion, the average postoperative flexion angle of the model was equivalent to those of other types of mobile bearing TKAs [2,9,13]. Postoperative flexion angle is supposed to have a good correlation with preoperative flexion angle. Additionally, it is influenced by a variety of factors, such as implant design and operative techniques. Therefore, it might be difficult to judge how much the model contributed to postoperative flexion angle. As to this model, only biomechanical laboratory study on human cadaveric specimens to assess cortex strain on the anteromedial aspect of the proximal tibia and tibial torsion from axial and rotational loading has suggested that the mobile- bearing prosthesis potentially reduced torque in the proximal tibia during knee rotation compared with the fixed- bearing prosthesis [14].

Dislocation of the polyethylene insert after Scorpio mobile-bearing TKA has been described previously [15]. In general, malpositioned components, flexion-extension gap mismatch, and laxity of soft tissue tension between the femoral and tibial surfaces can cause dislocation of a mobile bearing polyethylene insert [16,17]. In our case, the tension and alignment was considered to be adequate. Our case and duplicated saw bone model demonstrated that failure of the locking system resulted in the dislocation of the insert [15]. Dislocation of polyethylene insert is rare and it can easily be overlooked at the early stage [18,19]. Severe damage to either the femoral or tibial component might occur if there was delay in treatment. When a patient who has undergone TKA complains of pain, swelling, sudden instability, clicking, or locking sensation, it is important to consider insert dislocation as a possible explanation for these symptoms.

Sledge et al. [20] have reported that patients with RA achieve outcomes similar to those of patients with OA with regard to range of motion and pain relief after TKA. Similarly, we found no significant differences between two groups with regard to the clinical results and the radiographic results, although there was significant improvement of the JOA score in both groups. The Norwegian Arthroplasty Register [21] have shown that the cumulative 5- year survival of TKAs was 98.9% in RA patients and 99.3% in OA patients, with revision for infection as the end point. It means that the risk of revision of primary TKAs was statistically higher in RA patients than in OA patients. Therefore, longer follow- up evaluation is necessary to compare results of OA patients and RA patients.

There are some limitations to this study. First, the follow up rate is not adequate for the mid- term results. Although improved follow- up rate might bring different clinical and radiographic results, the number of cases is supposed to be still adequate. Second, there are no control groups in this study. We should have compare results of the design with those of other types of a mobile- bearing TKA.

In conclusion, there was significant improvement with regard to the clinical and radiographic results of the Scorpio mobile- bearing knee model as well as other types of mobile TKA. The risk of polyethylene insert dislocation related to the mobile bearing TKA is a cause for concern.

## Competing interests

The authors declare that they have no competing interests.

## Authors’ contributions

HK, NM, NT, and YA participated in the design of the study. YM, MA, HO, KI, KH, and TI conceived the study, and participated in its design and coordination. HK, NM, YM, NT, YA, MA, HO, KI, KH, and TI performed operation. HK and TS drafted the manuscript. All authors read and approved the final manuscript.
